# Effective Coupling of Light Emitting Diode to a Commercial Capillary Electrophoresis Laser-Induced Fluorescence Instrument for High-Sensitivity Analysis of Fluorescently Labeled Glycans

**DOI:** 10.1021/acs.analchem.5c05248

**Published:** 2025-10-17

**Authors:** Filip Duša, Pavlína Dadajová, Jozef Šesták, Jana Lavická

**Affiliations:** † Institute of Analytical Chemistry of the Czech Academy of Sciences, Veveří 967/97, Brno 602 00, Czech Republic; ‡ Department of Chemistry, Faculty of Science, Masaryk University, Kamenice 753/5, Brno 625 00, Czech Republic

## Abstract

Light-emitting diodes offer a low-cost, power-efficient, and compact solution for fluorescence excitation in analytical instrumentation. This study discusses the coupling of a near-ultraviolet light-emitting diode (340 nm) to a commercial capillary electrophoresis instrument and presents two feasible strategies: a simple, robust, and low-cost option with moderate efficiency and a more complex but significantly more efficient design for applications demanding maximum sensitivity. The coupling efficiency was assessed using capillary zone electrophoresis of maltooligosaccharides labeled with the UV-excitable fluorophore, 6-[4-(4-methylpiperazin-1-yl)­phenyl]­pyridine-3-carbohydrazide. Compared to a commonly used indirect light guide coupling approach, the new direct coupling design, incorporating a single ball lens, provided a 10.7-fold increase in the fluorescence signal. The design incorporating two plano-convex lenses increased the fluorescence signal by a factor of 31.2 and achieved limits of detection between 99 and 105 nmol/L for the analyzed labeled maltooligosaccharides. This optimized configuration enabled the successful *N*-linked glycan analysis from minute sample quantities, specifically, 28.8 ng of ovalbumin and 7.49 ng of ribonuclease B.

## Graphical abstract

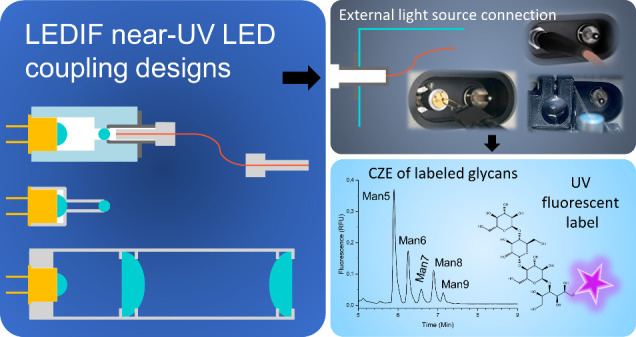

## Introduction

Laser-induced fluorescence (LIF) detection is a highly sensitive detection technique used in capillary electrophoresis (CE) for the analysis of a wide variety of molecules, including proteins, nucleic acids, and small molecules^1,2^ to solve many modern analytical challenges in fields ranging from biotechnology and pharmaceuticals to environmental analysis. The primary advantage is its outstanding sensitivity, which can be several orders of magnitude greater than UV–vis absorbance detection. This benefit arises from the fundamental difference between absorbance and fluorescence measurements. The photosensitive element of the absorbance detector monitors relatively high-intensity light levels and its slight changes. Due to relative nature of absorbance, the only instrumental parameter influencing the absorbance signal and sensitivity is the optical path length, which can be extended up to 10-fold in a CE instrument (e.g., when detection in a 75 μm I. D. capillary is replaced by sensitive G1600–60027 high sensitivity cells used in Agilent CE instrumentation). On the other hand, effective separation of excitation and emission light in a fluorescence detector ensures that the photosensitive element collects only the light emitted by the analyte itself, against a dark background. That way, the background noise is way lower, giving a much higher signal-to-noise ratio. Furthermore, the fluorescence signal is directly proportional to the excitation light intensity, which can be increased by several orders of magnitude. This allows the highly sensitive analysis of minute sample volumes and makes it the preferred method for detecting analytes at very low concentrations, often reaching the nanomolar to picomolar range.^3^

Often, the analyte of interest is not fluorescent, requiring the attachment of a fluorescent tag to enable detection.^2,4,5^ The choice of labeling agent determines the required hardware configuration. Specifically, the laser source must be selected to match the maximum excitation wavelength of the chosen fluorescent tag to ensure efficient excitation and a strong fluorescent signal. Critical considerations for constructing sensitive LIF systems include enhancing the fluorescence signal-to-noise ratio and considering the optical configuration of detection (e.g., confocal detection, 90° on-capillary detection, and sheath-flow detection).^6^ Recent developments in the field have been described by Šesták et al.^3^

Commercially available CE/LIF instruments have a limited number of excitation wavelength configurations. However, researchers often need a selection of different excitation wavelengths for a broad range of different compounds and new fluorescent labels. Regarding glycan analysis, for example, the standard setup using the 488 nm laser works exceptionally well with the most commonly used fluorescent tag, 8-aminopyrene-1,3,6-trisulfonic acid (APTS). However, due to the recent development of new, alternative fluorescent labels with different spectral properties, modification of this setup becomes essential.

For this purpose, the commercial CE/LIF systems typically allow the user to connect an external excitation source even if the system includes a built-in internal laser source. For example, Sciex CE/LIF instruments (PA800 or P/ACE MDQ Plus), one of the most common CE/LIF systems, can be equipped with the internal 488 nm laser module. An external laser or other excitation source can be coupled to the instrument via a fiber optic connector, and when a beamsplitter is installed in the detector, even dual channel detection is feasible. Furthermore, other commercially available fiber-coupled external lasers (e.g., a 532 or 561 nm laser) can be used for fluorescence excitation. The Zetalif LASER or LED induced fluorescence detectors from Adelis (https://www.adelis-tech.com/product-zetalif-detectors/) are good examples of a detector designed for coupling with CE or LC instruments, utilizing replaceable optical filters and light emitting diodes (LEDs) or lasers.

Successful adaptation of commercial CE/LIF systems for specialized applications^6^ requires the installation of appropriate optical filters and the effective coupling of an external light source. Selecting the right optical filters is crucial to ensuring that only the desired fluorescence emission is detected, thereby improving the signal-to-noise ratio. Effective coupling of an external light source ensures high intensity of excitation light at the point of detection, thus producing a high signal. Coupling of the external source can be easily achieved using an optical cable and standard fiber connectors or alternatively, custom-designed adapters (e.g., a fiber coupler) can be used. These adapters are often created using custom-machining or 3D printing.^7^ Recently, we have presented an adaptation of the LIF setup to the specific fluorescence spectra of 2-aminoacridone labeled human milk oligosaccharides, connecting an external solid-state laser with a wavelength of 405 nm to the CE instrument via a simple, 3D-printed laser-to-light-guide adapter.^8^ This setup can be used as a model for coupling different lasers to the commercial CE instruments.

Although the laser sources are preferred for LIF detection, LEDs are an alternative source for fluorescence excitation.^3,9,10^ LEDs offer an excellent opportunity to tailor the system for specific research needs beyond standard applications, mainly due to their lower cost, widespread availability, long lifetime, and low maintenance. They offer precise and stable intensity control and can be rapidly switched for multicolor imaging experiments. However, LEDs have a relatively broad emission peak, that necessitates use of additional filters to minimize crosstalk between different fluorophores, and they can be less efficient at exciting the target dye. Their light output is also highly divergent, requiring careful optical design to maximize collection efficiency. Additionally, LEDs are very efficient in the visible light spectrum region, with a higher plug wall efficiency (PWE). Blue LEDs have PWE of 80%, whereas UV LEDs have a significantly lower external quantum efficiency (PWE below 10%) due to the properties of the AlGaN-based semiconductor materials used.^11^

In this work, we present an alternative approach of coupling an LED-based external excitation source to a commercial CE/LIF system. Instead of the traditional indirect coupling using an optical patch cable, we performed direct illumination of the instrument’s port for the SubMiniature A (SMA) 905 connector. Two different designs, differing in complexity, are presented and discussed.

## Experimental Section

### Chemicals and Materials

All chemicals were at least of ACS or HPLC gradient grade purity quality. Formic acid (85%), methanol, and sodium hydroxide were obtained from Avantor (Randor, PA, USA). Acetic acid, acrylamide, ammonium persulfate, 3-(trimethoxysilyl)­propyl methacrylate, *N,N,N’,N’*-tetramethylenediamine, maltotetraose, maltopentaose, maltohexaose, bovine ribonuclease B (RNase B), chicken albumin (ovalbumin, OVA), and peptide-*N*-glycosidase F (PNGase F) were obtained from Merck (Prague, Czech Republic). The deionized water used in all experiments was purified by a Purite Select Neptune system (Watrex, Prague, Czech Republic). Fused silica capillaries were purchased from Molex (Lisle, IL, USA). Amicon Ultra centrifugal filters (3 kDa MWCO) were also purchased from Merck.

### Instrumentation

The P/ACE MDQ Plus system (Sciex, Brea, CA, USA), equipped with the original LIF detector, was used in this work. The LIF detector includes excitation and detection modules (SI1, Figure S1). The excitation module contains an in-built 488 nm laser (switched off during experiments) and the SMA (SubMiniature A) fiber port (for SMA905 connector) for an external light source connection. The detection module includes mounts for dichroic mirror, optical filters, and two independent photomultiplier tubes (PMT). In this work, no dichroic mirror, 370 nm long-pass filter (PN 66038, Edmund Optics, York, UK), and only single PMT were used (SI1, Figure S1). The PMT was original, unmodified, installed by the manufacturer.

The 340 nm LED (0.8 mW) (DUV340-HL46N, Roithner LaserTechnik, Vienna, Austria) was used as an external excitation source. Even though the manufacturer states the beam angle of 6° in the datasheet, the real observed angle of the light cone was approximately 20°. The LED was connected to an adjustable, constant current, DC-DC step-down module XL4015, powered by a regular 12 V DC adapter (both from a local DIY electronics supplier). The module was set to supply a constant current of 30 mA (40 mA is the maximum forward current listed in the datasheet supplied with the LED). The LED was coupled to the P/ACE MDQ Plus system’s SMA fiber port utilizing a variety of lab-designed adapters (Figure 1). The first design (Figure 1A) contained a 3D-printed mount for SMA connector (PN HASMA, Thorlabs, Newton, NJ, USA), 3.0 mm fused silica ball lens (PN 67384, Edmund Optics), and LED. The fiber patch cable (PN M112L01, Thorlabs) was used to connect SMA connector in the 3D-printed mount with the P/ACE MDQ Plus system’s SMA fiber port. The second design (Figure 1B) utilized a 3.0 mm fused silica ball lens (PN 67384, Edmund Optics) and an aluminum body (made in-house, SI1, Figure S2) for direct illumination of the P/ACE MDQ Plus system’s SMA fiber port.

**Figure 1:**
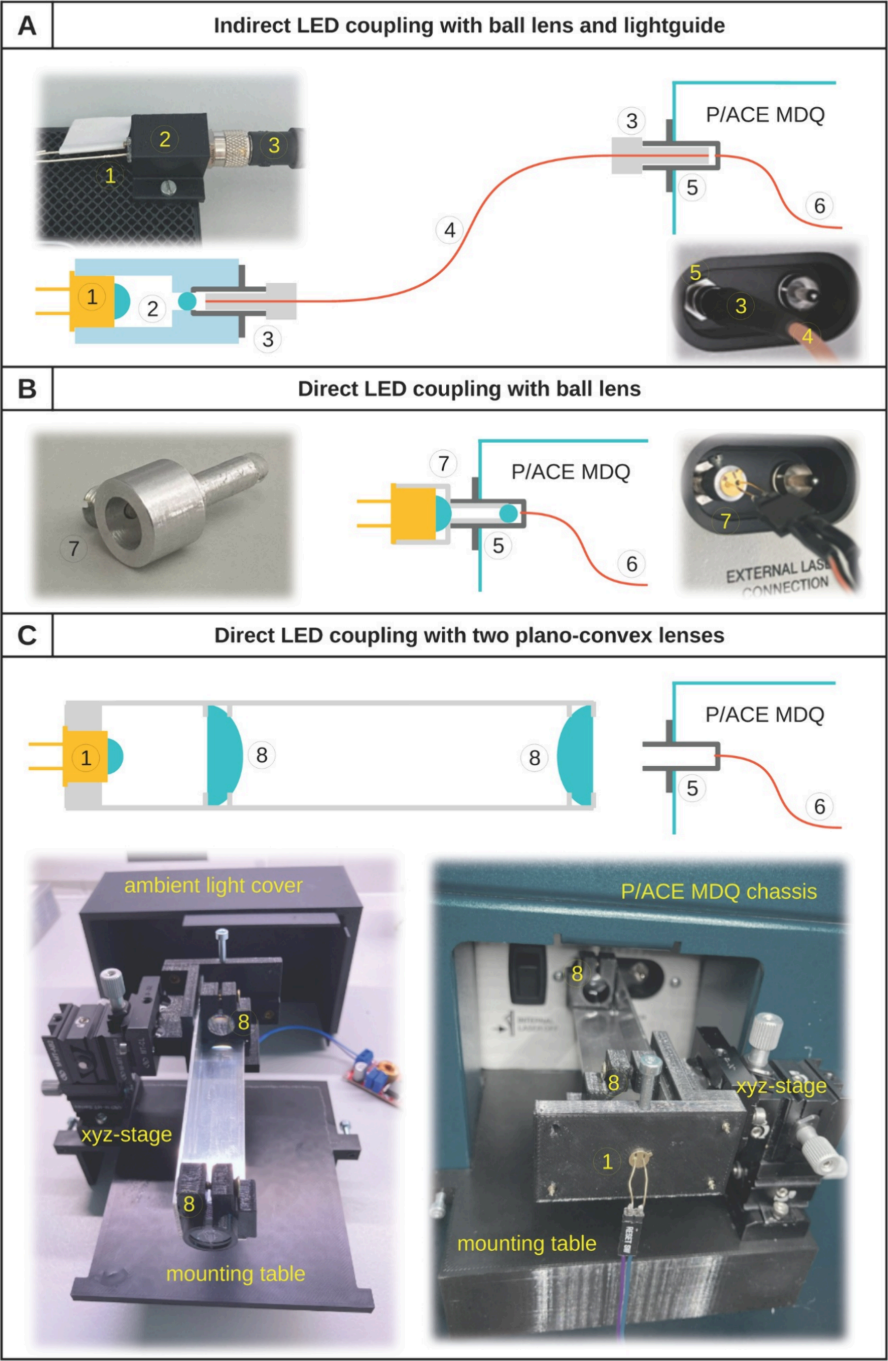
Schemes and photographic details of coupling LED to the P/ACE MDQ Plus; (A) indirect LED coupling with a 3 mm ball lens and lightguide; (B) direct LED coupling with a 3 mm ball lens; (C) direct LED coupling with two 12.5 mm plano-convex lenses; 1 – 340 nm LED; 2 – 3D-printed housing including LED, 3 mm ball lens, and SMA connector; 3 – SMA connection; 4 – standard optical cable; 5 – instrument’s SMA fiber port; 6 – optical fiber within P/ACE MDQ Plus LIF module; 7 – SMA905 connector compatible lathed aluminum housing including LED and 3 mm ball lens; 8 – 12.5 mm plano-convex lens with focal length of 19.9 mm.

The last design (Figure 1C) included two N-BK7 borosilicate crown glass, plano-convex lenses with a focal length of 19.9 mm and an outer diameter of 1/2 in. (PN LA1074, Thorlabs). The compact dovetail linear stage (M-MT-XYZ, Newport Corporation, Andover, MA, USA) mounted on 3D-printed table was utilized for precise alignment of the focal point. The fluorescence excitation and emission spectra of the label were measured on a JASCO FP-8500 fluorescence spectrometer (ABL&E-JASCO, Vienna, Austria). Source files (3mf files) used for 3D-printing of components are enclosed as Supporting Information (SI2–SI8).

### Maltooligosaccharides and *N*-Linked Glycans Labeling

Standards of maltotetraose, maltopentaose, and maltohexaose (DP4–6) were labeled using a recently developed UV fluorescent label, 6-[4-(4-methylpiperazin-1-yl)­phenyl]­pyridine-3-carbohydrazide (DMPPP hydrazide), via a hydrazide reaction protocol.^12^ As this technical note focuses exclusively on the technical solution of the LED source coupling to a CE instrument, the label synthesis, properties, and characterization will be presented separately elsewhere. In summary, a solution of 0.01 M DMPPP hydrazide in methanol containing 10% (v/v) acetic acid (50 μL) was added to the dried equimolar mixture of maltooligosaccharide standards, with a molar maltooligosaccharide to label ratio of 1:10. The labeling was performed at 50 °C for 18 h. The labeled samples were dried in a vacuum concentrator and stored in the freezer before further analysis. *N*-linked glycans were released from glycoproteins (RNase B, OVA) using PNGase F, according to the previously published protocol.^13^ The released glycans were purified using the Amicon filters, following the manufacturer’s protocol, and then dried in a vacuum concentrator. The labeling of dried glycans with DMPPP hydrazide was carried out using the same procedure. Due to the low excess of the label, the labeled samples were analyzed without any purification step, which often causes sample loss. Alternatively, the purification step can also be omitted when using a properly optimized background electrolyte.^12,14^

### CE/LED-Induced Fluorescence

CE/LED-induced fluorescence (CE/LEDIF) analyses were performed in fused silica capillaries with an inner diameter of 50 μm, a total length of 40 cm, and an effective length of 30 cm. The inner wall of the capillaries were coated with linear polyacrylamide (LPA) according to a previously published procedure.^15^ A background electrolyte (BGE) was prepared by mixing a 1 M formic acid solution with methanol in a 1:1 (v/v) ratio. The capillary was rinsed with BGE at 2.1 bar (30 psi) for 3 min before each injection. Sample was injected at 34.47 mbar (0.5 psi) for 15 s. A separation voltage of 30 kV was applied across the capillary during the capillary zone electrophoresis (CZE) analysis, with a typical run time below 20 min. LIF detector was set to dynamic range of 10 RFU, with filter settings set to “normal” and recording rate of 2 Hz. Electropherograms were recorded using the 32 Karat software (Sciex) and further processed in OriginPro 2025 (OriginLab, Northampton, MA, USA).

## Results and Discussion

The successful implementation of new fluorescent labeling in CE is fundamentally dependent on the precise adjustment of the detection system to match the unique spectral properties of the labeling used.^4^ Recently, a novel phenylpyridine-based fluorescent tag, DMPPP hydrazide, was designed and synthesized. This new fluorescent tag, having a maximum of fluorescence excitation at 325 nm (Figure 2), was used in this work. Based on the excitation range in the near UV region, the commercial CE/LIF system equipped with a 488 nm laser module required technical modifications, particularly with regard to the selection of a suitable excitation source. Given the planned modification of the label using additional substituents that alter the fluorescent properties, as well as a broader applicability of the developed instrumentation, an inexpensive, simple, and easily exchangeable LED excitation source was selected. A 340 nm LED was used for excitation of DMPPP hydrazide labeled analytes.

**Figure 2:**
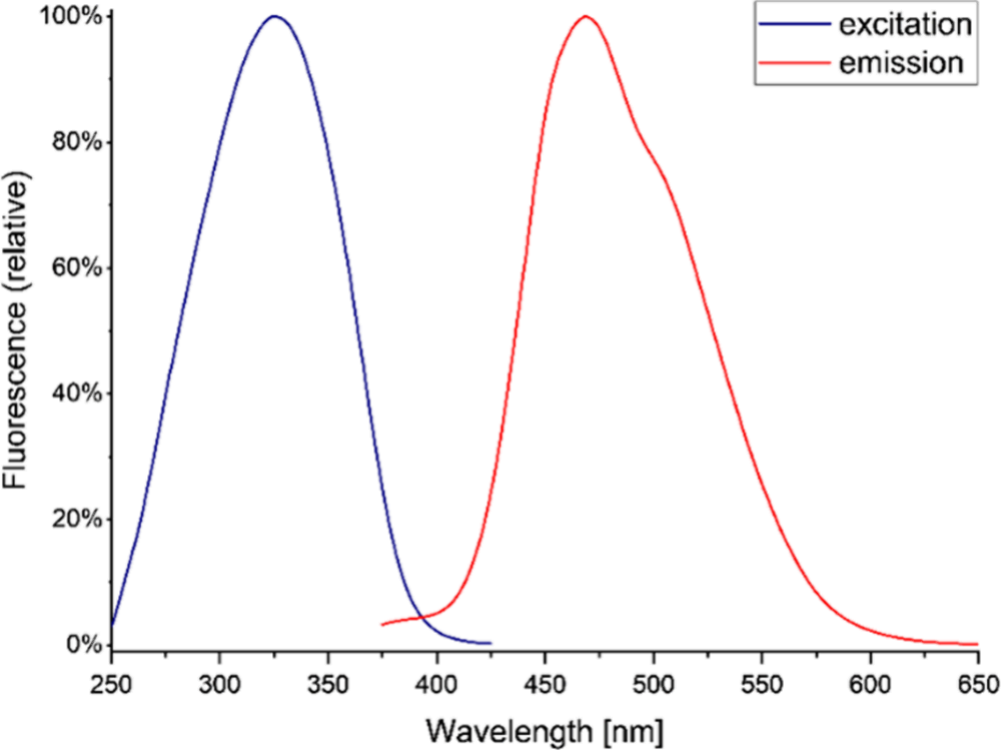
Normalized excitation and emission spectra of the 1 μmol/L DMPPP hydrazide label dissolved in a solution containing 50% (v/v) 1 M formic acid and 50% (v/v) methanol. The excitation spectrum was measured at 470 nm in the range of 250–450 nm with 1 nm steps and the emission spectrum was measured at 325 nm in the range of 350–650 nm with 1 nm steps. A 10 mm rectangular cell cuvette was used for the measurements.

Three different setups were developed to couple an external LED excitation source to the LIF detector module. Based on our previous work,^8^ a simple 3D-printed adapter was used to accommodate the LED, which was axially coupled to the lightguide by a 3 mm ball lens (Figure 1A, files for 3D printing). This configuration is hereinafter called “indirect LED coupling with 3 mm ball lens and lightguide”. However, a very weak fluorescence signal was observed during the CZE analysis of the three maltooligosaccharide standards labeled with DMPPP (Figure 3A). This demonstrates that the setup which normally provides satisfactory coupling of high-power lasers (>20 mW) to the commercial CE/LIF system,^8^ is barely usable for a low power excitation light source, such as the 0.8 mW UV LED used in this study. Light losses occurring at the lightguide connection points can be the possible reason for this unsatisfactory LED coupling. Therefore, the other two tested designs of LED coupling implement a direct illumination of the commercial CE/LIF system’s SMA fiber port.

**Figure 3:**
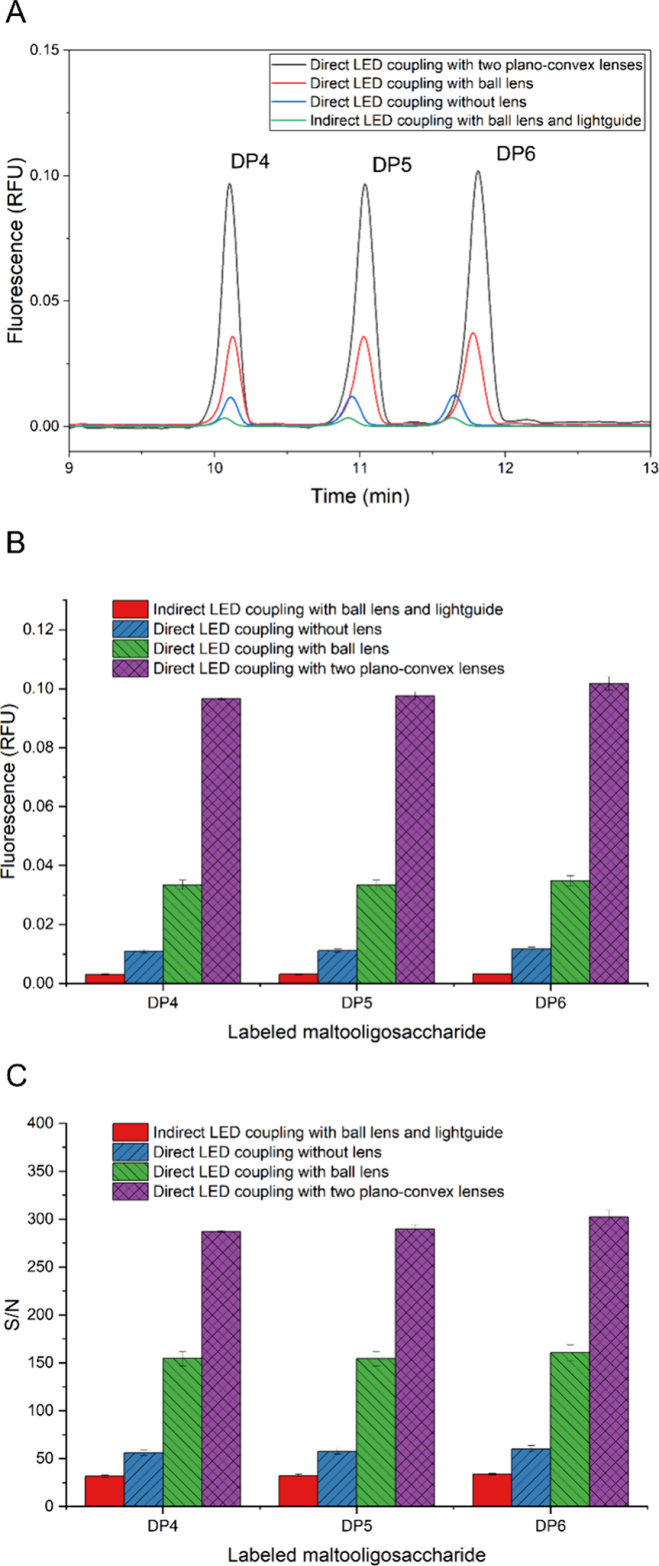
(A) CZE analysis of DMPPP-labeled maltooligosaccharides; separation conditions: 50 μm LPA coated fused silica capillary (30/40 cm length (eff/tot)), BGE: a mixture of 1 M formic acid and methanol in a 1:1 (v/v) ratio, sample injection at 34.47 mbar for 15 s, 30 kV separation voltage for 20 min, LIF detector dynamic range of 10 RFU, “normal” filter settings, and recording rate of 2 Hz. Due to continual shift caused by LPA coating and organic BGE, the CZE records were aligned for better visibility using Origin Pro multiplier function (values: 1; 1.04; 1.115; 1.19). (B) Fluorescence peak height of DMPPP labeled maltooligosaccharides and (C) S/N ratios of particular LED coupling.

The first tested direct LED coupling design (Figure 1B) consists of an aluminum body (SI1, Figure S2), integrating an LED holder and a tubular protrusion of a 3.2 mm outer diameter, which corresponds to the diameter of the SMA905 connector. The inner bore of the protrusion measures 2.4 mm. This configuration is hereinafter called “direct LED coupling without lens”. The fluorescence intensity (height) of the DMPPP-labeled maltooligosaccharide peak detection increased 3.5 times (Figure 3B), and the signal-to-noise (S/N) ratio increased 1.8 times (Figure 3C) in comparison to the indirect LED coupling with a 3 mm ball lens and lightguide.

In the next step, the 3 mm ball lens was attached to the top of the protrusion with 5 min epoxy glue (Figure 1B). This configuration is hereinafter called “direct LED coupling with ball lens”. The ball lens partially focused the light to the center of the instrument’s SMA fiber port (SI1, Figure S3) and further improved the observed peak height by a factor of 3 and the S/N ratio by a factor of 2.7. Compared to the indirect LED coupling with ball lens and lightguide, the observed peak height increased by a factor of 10.7, and the S/N ratio increased by a factor of 4.8. This demonstrates that utilizing such a simple device can already boost the amount of excitation light transferred to the system by 1 order of magnitude. Considering fundamental limitations of this very simple optical system, like a small aperture of the 3 mm ball lens, a high angle of focused beams vs small acceptance angle of the instrument’s internal lightguide (SI1, Figure S3), a further gain in the signal should be feasible with somewhat more advanced optics.

Therefore, a coupling setup featuring two plano-convex lenses (with a diameter of 12.5 mm and a focal length of 19.9 mm) was developed (Figure 1C). The first lens collimates the light cone of the LED, and the second lens focuses the collimated light into the instrument’s SMA fiber port (SI1, Figure S3). The distance between the LED and the focal point was approximately 10 cm. This configuration is hereinafter called “direct LED coupling with plano-convex lenses”. The stand carrying the optics was made of an aluminum l-profile (20 × 20 × 1.5 mm), with 3D-printed parts for mounting the LED and lenses. It was mounted on an XYZ linear stage to enable precise positioning of the focal point. Finally, a platform supporting the complete setup was designed and 3D-printed to fit the XYZ-stage and the optics stand. The platform was placed on the sliding door, and two M3 screws were used to secure it firmly against the platform pins inserted into the inner side of the door chassis. Due to the open construction of the system, a box covering the complete LED coupling setup was manufactured to block out ambient light (experiments were performed in a regular lab during daylight). The files for 3D-printing are provided as Supporting Information (SI4 to SI8).

While the 370 nm long-pass filter blocked most of the 340 nm LED-emitted spectrum, a small fraction of the longer wavelengths caused some increase of the baseline level. Monitoring of the baseline level related to the movement of the stand in the particular axes was used to find the optimal position of the optical system in front of the instrument’s SMA fiber port (SI1, Figure S4, for a record of the baseline changes in connection to the axes change).

Finally, a mixture of DMPPP-labeled maltooligosaccharides was analyzed using CZE and the direct LED coupling with plano-convex lenses (Figure 3A). The results confirmed a significant increase in the detected fluorescence peak heights, surpassing the indirect LED coupling with ball lens and lightguide by a factor of 31.2 and the S/N ratio by a factor of 8.9. Compared to the direct LED coupling with ball lens, a 2.91-fold signal (1.9-fold S/N) increase was accomplished. However, this came at the cost of the simplicity and robustness of the coupling system.

The observed differences in the performance of the tested designs clearly reflect the varying effectiveness of light transmission from the LED to the SMA fiber port of the commercial CE/LIF system. In the indirect LED coupling with ball lens and lightguide, the losses of light occur at both ends of the optical cable (due to reflection, dispersion and numerical aperture limitations) and partially within the lightguide (attenuation). The direct LED coupling with ball lens clearly eliminates these light losses as it can easily increase the observed peak height by a factor of 10. Finally, the additional signal gain observed with the more complex direct LED coupling with plano-convex lenses clearly indicates the highest excitation light throughput among the tested designs.

The CE/LEDIF analysis employing the direct LED coupling with plano-convex lenses was also evaluated in terms of limits of detection (LODs). The LODs of DMPPP-labeled maltooligosaccharides were 104.5, 103.5, and 99.2 nmol/L for DMPPP-labeled DP4, DP5, and DP6, respectively (S/N = 3, n = 3). This is comparable with recently reported LODs ranging from 0.1 to 0.3 μmol/L for LEDIF detection of pterins using a commercial setup of Zetalif LEDIF detector (λ_ex_/λ_em_ = 365/450 nm).^16^ Approximately 5 μmol/L LOD (recalculated) for phenylglyoxal hydrate-labeled tryptophan was achieved using a custom-designed optical setup for an in-house fabricated CE (2 mW, 380 nm LED with a detector using microscope optics).^17^

Lower LODs can be reached when the detectors are designed as stand-alone and high-power Vis LEDs are utilized. For a system that used a standalone LEDIF detector for CE together with a high-power LED (470 nm, 170 mW), a LOD of up to 800 pM was achieved for APTS-labeled maltooligosaccharides.^18^ A high-power, single-color blue LED (3 W) was used for a CE detector with LODs of 613 ± 13 pM for fluorescein dye.^19^ A similar approach using a high-power blue LED (120 mW) in a standalone multi detector (contactless conductivity, ultraviolet absorbance, and LEDIF) resulted in an LOD of 10 nmol/L.^20^ More recently, a multi detector design reported LOD of 15 nM for fluorescein isothiocyanate labeled bovine serum albumin, however, it could be improved to LOD of 0.8 nM if used as a standalone LEDIF detector.^21^ The newly designed coupling system is comparable to or more sensitive than the current UV LED based detector designs. Development and availability of high efficiency UV LEDs could bring further improvement to compete with high-power blue LED designs.

To demonstrate the feasibility of this setup for glycan analysis, *N*-linked glycans released from RNase B and OVA were also labeled with DMPPP and analyzed using the direct LED coupling with plano-convex lenses. Figures 4A and 4B show the obtained electropherograms of DMPPP-labeled *N*-linked glycans. Although the well-known pattern of RNase B *N*-linked glycans allows peak assignments, the identification of individual glycans released from OVA requires further optimization of the separation conditions and/or their identification in a combination with MS detection. In terms of sensitivity of DMPPP-labeled glycan analysis, the detected peak signals correspond to the original protein concentrations of 10 mg/mL for OVA and 2.6 mg/mL for RNase B, before the N-glycan cleavage and labeling. Considering the injected sample volume, the electropherograms show analysis of labeled glycans from 28.8 ng of OVA and 7.49 ng of RNase B. For comparison, smartphone technology-based CE/LEDIF was used to analyze APTS-labeled IgG *N*-linked glycans at a protein concentration of 0.2 mg/mL, with sample fluorescence enhanced by advanced signal processing.^22^

**Figure 4:**
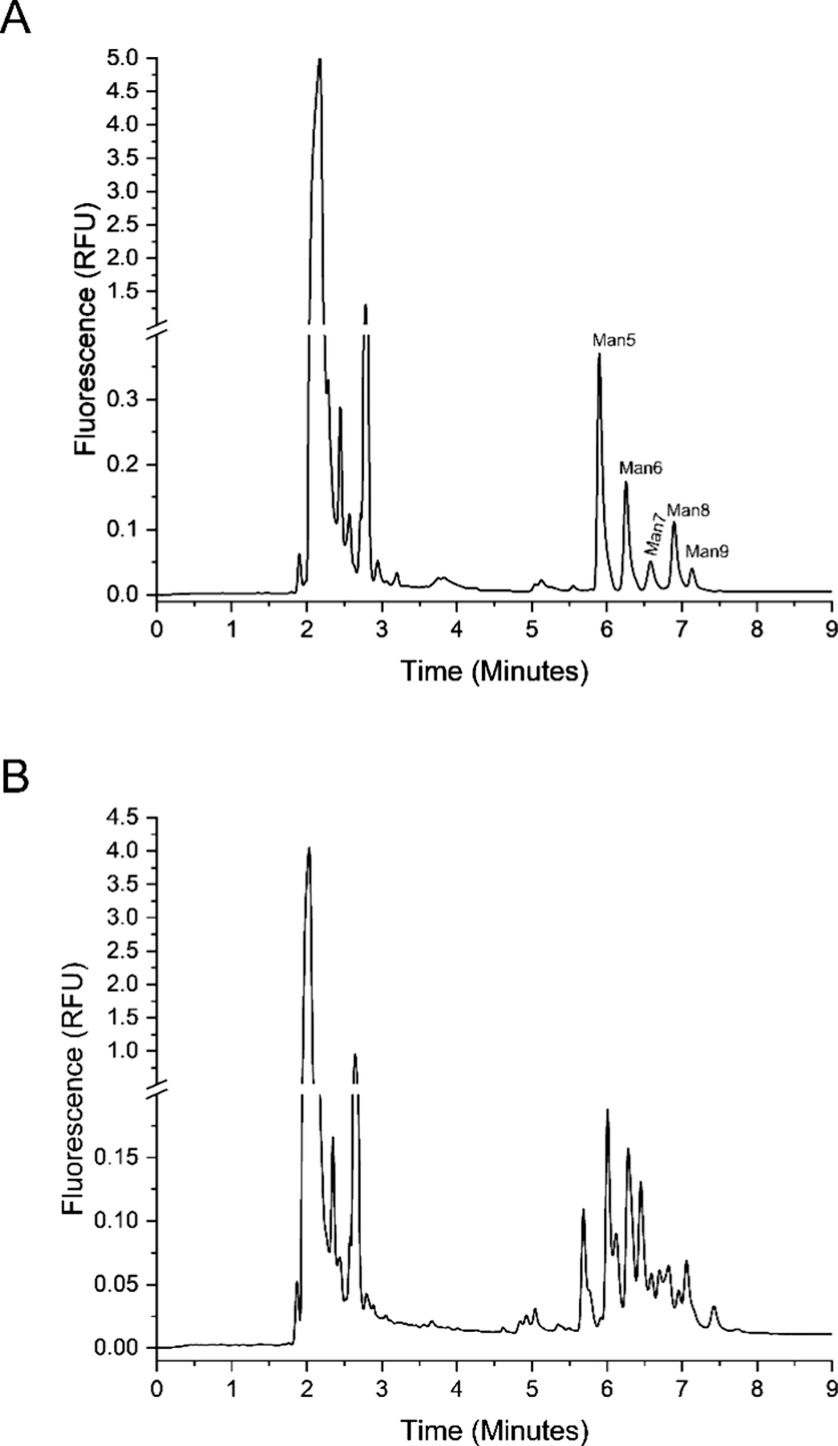
CZE analysis of DMPPP-labeled *N*-linked glycans released from (A) RNase B and (B) OVA using direct LED coupling with plano-convex lenses. Separation conditions: 50 μm LPA coated fused silica capillary (30/40 cm length (eff/tot)), BGE: a mixture of 1 M formic acid and methanol in a 1:1 (v/v) ratio, sample injection at 34.47 mbar for 15 s, 30 kV separation voltage for 20 min, LIF detector dynamic range of 10 RFU, “normal” filter settings, and recording rate of 2 Hz.

## Conclusions

Essentially, modifying the LIF detection setup on the commercial CE instrument is a feasible approach to expand the limits of detection, sensitivity, and application scope. Our investigation shows that connecting a near-UV LED to this commercial CE instrument is a feasible modification for applications where the excitation light flexibility is the primary objective rather than ultrahigh sensitivity detection. Our design also brings substantial cost-effectiveness, with about 150 € per LED source with direct insert coupling. This is a significant advantage compared to the usual budget of over 2000 € for boxed laser sources. This modification significantly broadens the universality of CE/LEDIF, as it requires minimal material and manufacturing cost, and the setup is very robust and simple to use.

Efficient coupling of the emitted light is essential to maintain system sensitivity. In this study, three coupling designs that differ in the level of complexity were compared. The designs were evaluated by CZE analysis of labeled maltooligosaccharides and *N*-linked glycans. While the most sophisticated design showed the highest fluorescence signal, comparable to or even more sensitive than current detector designs, the insert-based design offered the best ratio of design simplicity to signal enhancement. Overall, using LEDs as the excitation source is an excellent, easily adjustable (LED sources can be switched), and low-cost solution for detecting analytes that are present at moderate concentrations.

## Supplementary Material





## Data Availability

The data that support the findings of this study are openly available in the ASEP Data Repository at 10.57680/asep.0637926, reference number 0637926.

## Notes

Additional technical schemes and descriptions of the developed LEDIF detector setups (Figures S1–S3) and their optimization (Figure S4) (PDF)
Source files used for 3D printing of components of developed LEDIF detector setups (ZIP)

## Abbreviations

APTS: 8-aminopyrene-1,3,6-trisulfonic acid
BGE: background electrolyte
CE: capillary electrophoresis
CZE: capillary zone electrophoresis
DMPPP hydrazide: 6-[4-(4-methylpiperazin-1-yl)­phenyl]­pyridine-3-carbohydrazide
DP4: maltotetraose
DP5: maltopentaose
DP6: maltohexaose
I. D.: internal diameter
LED: light-emitting diode
LEDIF: LED-induced fluorescence
LIF: laser-induced fluorescence
LOD: limit of detection
OVA: chicken albumin (ovalbumin)
PMT: photomultiplier tubes
PNGase F: peptide-*N*-glycosidase F
PWE: plug wall efficiency
RNase B: bovine ribonuclease B
SMA: SubMiniature A
UV: ultraviolet
vis: visible
