# Supplementary material for: Effective Coupling of Light Emitting Diode to a Commercial Capillary Electrophoresis Laser-Induced Fluorescence Instrument for High-Sensitivity Analysis of Fluorescently Labeled Glycans
Source: Anal Chem. 2025 Oct 17;97(42):23032–9. doi: 10.1021/acs.analchem.5c05248 (PMC12573225; doi:10.1021/acs.analchem.5c05248)
Supplement: Supplementary file 1 [file ac5c05248_si_001.pdf]

Supporting Information

for Technical Note

Effective Coupling of Light Emitting Diode to a Commercial Capillary Electrophoresis Laser-Induced Fluorescence Instrument for High-Sensitivity Analysis of Fluorescently Labeled Glycans

Filip Duša[†], *Pavlína Dadajová*[†,‡], *Jozef Šesták*[†], *Jana Lavická*[†,*]

AUTHOR ADDRESS

† Institute of Analytical Chemistry of the Czech Academy of Sciences, Veveri 967/97, 602 00 Brno, Czech Republic

‡ Department of Chemistry, Faculty of Science, Masaryk University, Kamenice 753/5, 625 00, Brno, Czech Republic

*Corresponding Author

Dr. Jana Lavicka, Institute of Analytical Chemistry of the Czech Academy of Sciences, Veveri 967/97, 602 00 Brno, Czech Republic; (orcid.org/0000-0002-0218-8372); lavicka@iach.cz.

Table of Contents

Figure S1. CE/LIF scheme of the P/ACE MDQ Plus system and scheme of the detection module .. S2

Figure S2. Technical drawing of the LED insert S3

Figure S3. Ray tracing diagrams of direct LED coupling with a ball lens and plano-convex lenses..S4

Figure S4. LED XYZ-stage positioning LIF signal record S5

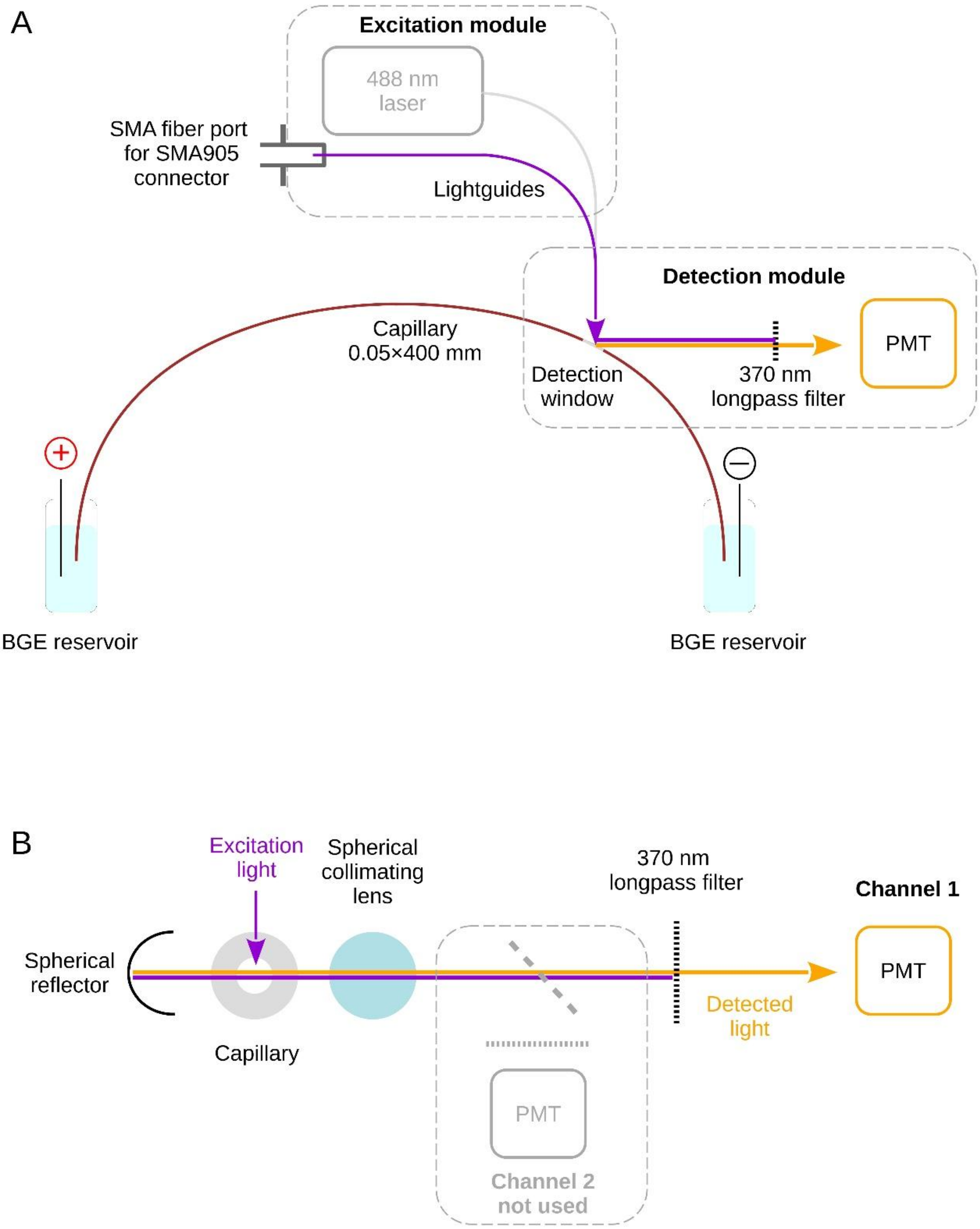

Figure S1. (A) CE/LIF scheme of the P/ACE MDQ Plus system. (B) Scheme of the detection module. In-built 488 nm laser was not used in this work. The system was used in a single channel (Channel 1) mode. Beam splitter was removed to maximize the amount of collected light in the Channel 1.

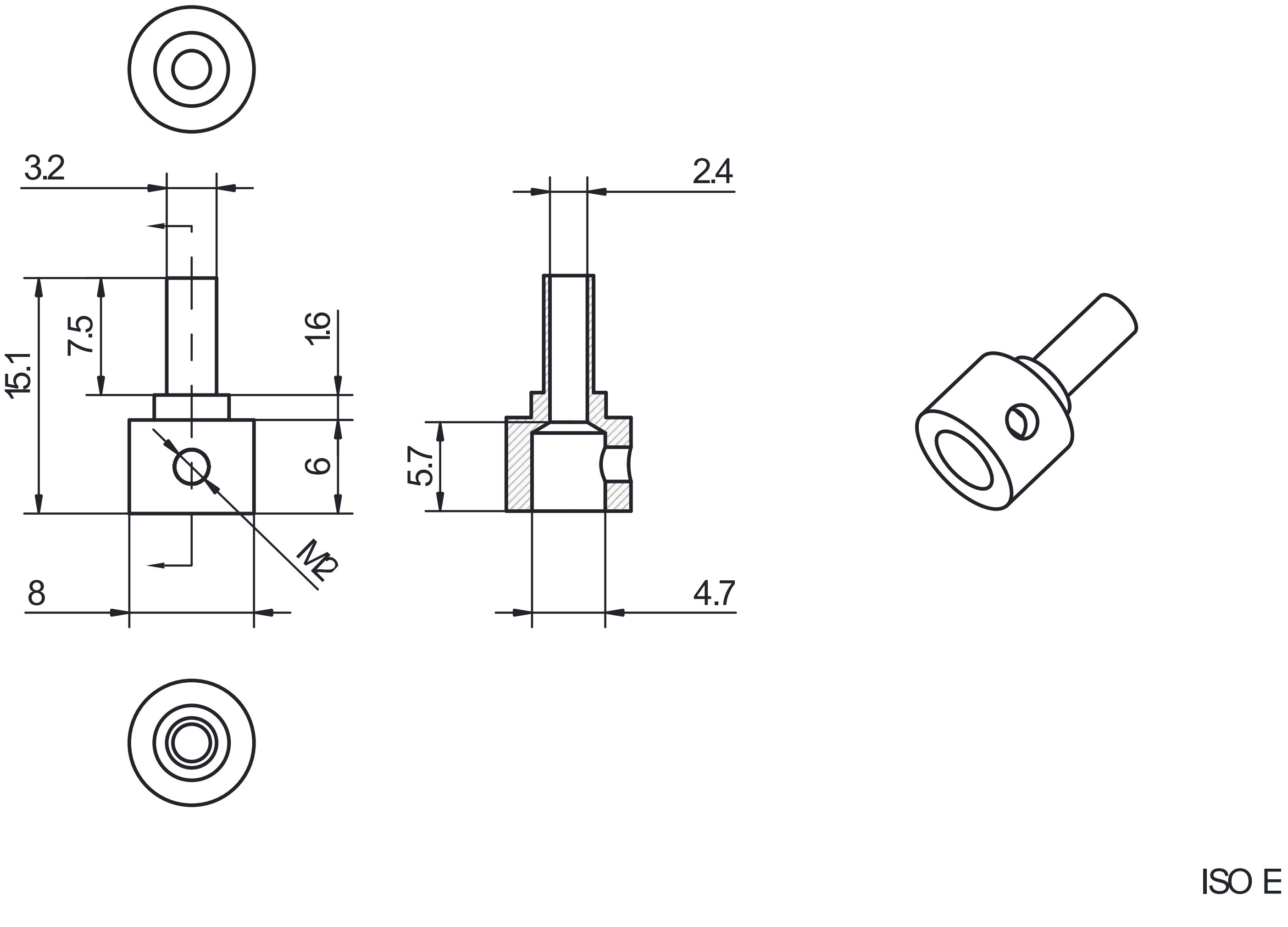

Figure S2. Technical drawing of the LED insert.

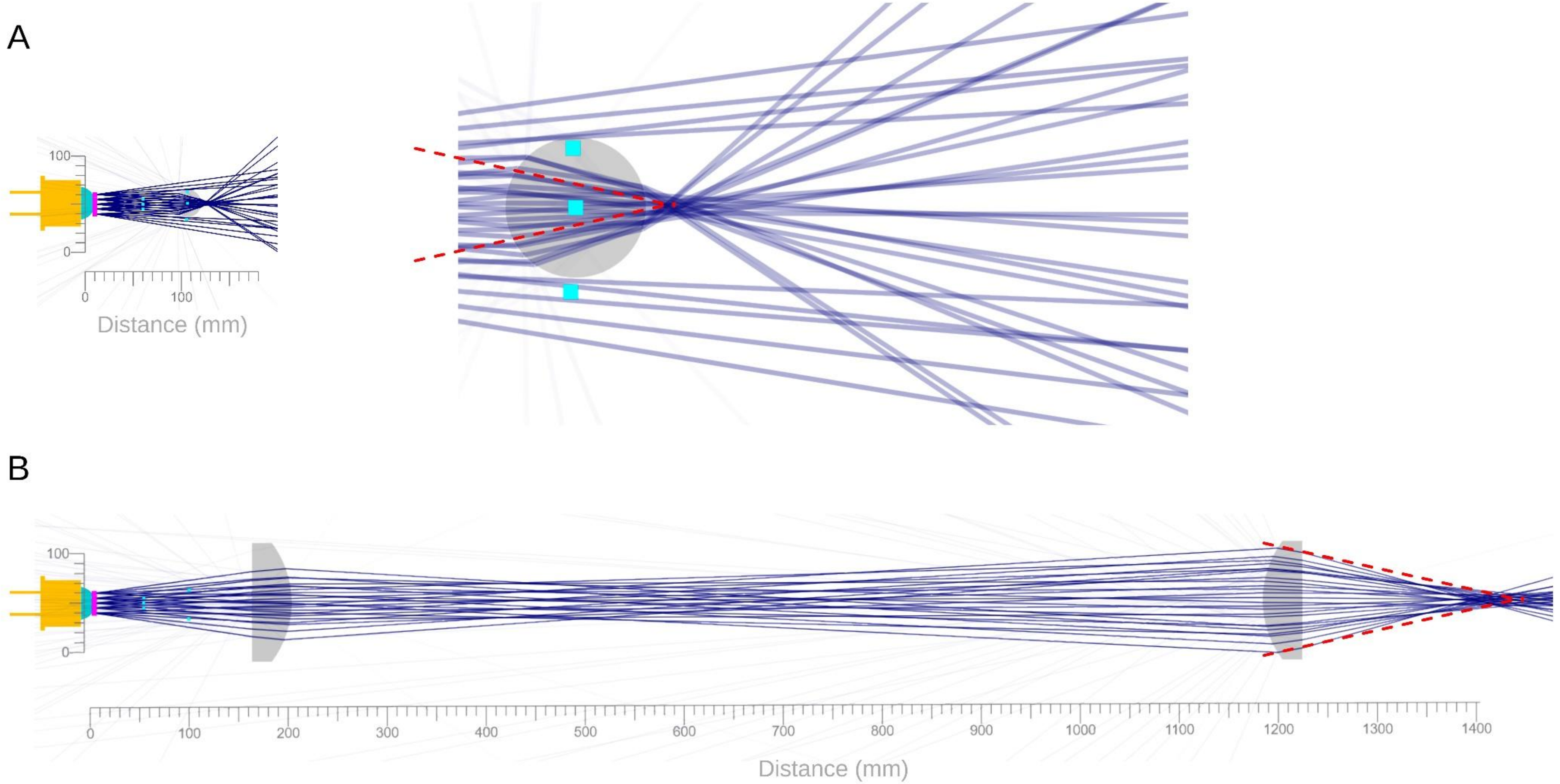

Figure S3. Ray tracing diagrams of (A) direct LED coupling with 3 mm ball lens and (B) direct LED coupling with two 12.5 mm plano-convex lenses. Red dashed lines indicate angle of 25° (0.22 NA lightguide´s acceptance angle multiplied by 2). Ray calculations were executed with the free web-based Ray Optics Simulation tool accessible in https://phydemo.app/ray-optics/. Refractive indices used for calculations were 1.48 for fused silica ball lens or 1.54 for N-BK7 glass plano-convex lenses.

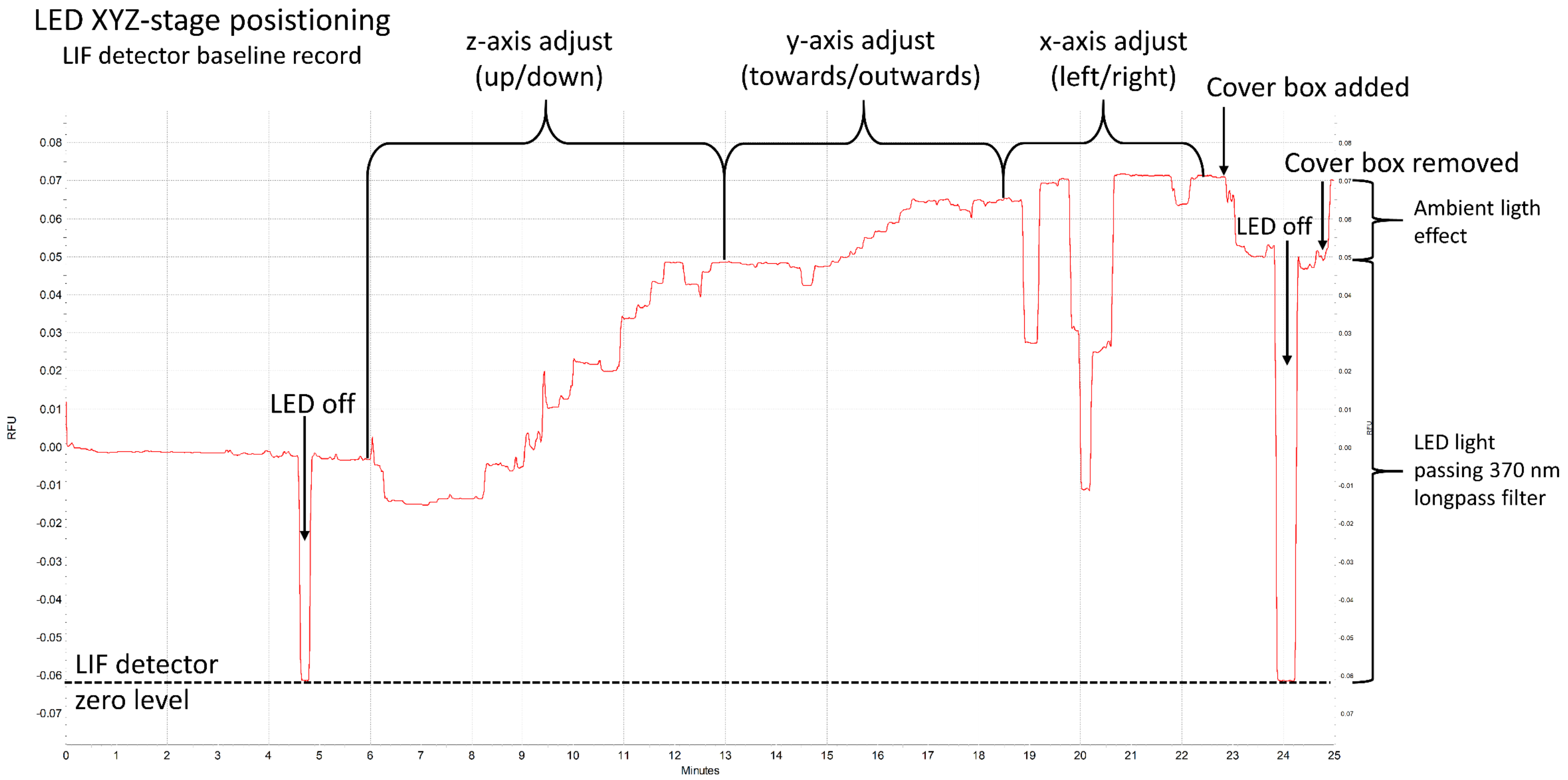

Figure S4. LED XYZ-stage positioning LIF signal record.
